# Thiolate–Protected Gold Nanoparticles Via Physical Approach: Unusual Structural and Photophysical Characteristics

**DOI:** 10.1038/srep29928

**Published:** 2016-07-18

**Authors:** Yohei Ishida, Ikumi Akita, Taiki Sumi, Masaki Matsubara, Tetsu Yonezawa

**Affiliations:** 1 Division of Material Science and Engineering, Faculty of Engineering, Hokkaido University, Kita 13 Nishi 8, Kita-ku, Sapporo, Hokkaido 060-8628, Japan

## Abstract

Here we report a novel physical approach for thiolate–protected fluorescent gold nanoparticles with a controlled size of the order of a few nanometers. This approach is based on a sputtering of gold into a liquid matrix containing thiolate ligand as a stabilizer at various concentrations, thus no reductant was used. The size of the gold nanoparticles was successfully controlled to range from 1.6 to 7.4 nm by adjusting the thiol concentrations. Surface plasmon absorption was observed in larger nanoparticles, but it was not observed in smaller ones. Such smaller nanoparticles fluoresced at around 670 nm with a small spectral shift according to their size, however, the diameter (1.6–2.7 nm) was very strange to show such red emission compared with photophysical characteristics of reported gold cluster or nanoparticles synthesized by chemical method. By detailed investigations using TEM, HAADF-STEM, XPS, and TGA, and size fractionation by size exclusion chromatography, we finally arrived at the plausible mechanism for the origin of unusual fluorescence property; the obtained gold nanoparticles are not single-crystal and are composed of aggregates of very small components such as multinuclear gold clusters or complexes.

Fluorescent gold nanoparticles (Au NPs) have been found to be useful in a wide variety of fields such as sensing, optoelectronics, cellular labelling, and imaging because of their unique optical properties and chemical stability^1^,^2^,^3^,^4^,^5^,^6^. As the fluorescence property of Au NPs is resulted from the quantum confinement effect^7^,^8^,^9^, this nanoscale characteristic is controlled by the size and shape of the nanomaterial.

One typical approach to prepare uniform Au NPs is the chemical reduction of Au^3+^ ions in a solution with a reducing agent such as NaBH_4_, hydrazine, alcohol, polyol, DMF, or citric acid^4^,^10^. Since naked metal NPs easily coalesce and change their size or shape, various organic molecules such as amines and mercaptants, or polymers such as PVP have been used as stabilizing reagents because of their good coordination properties with gold^2^,^4^,^10^. Chemical synthesis is very versatile in terms of controlling NP size and enables a variety of applications; however, it usually generates harmful byproducts and NPs with limited purity, especially owing to the use of reductants. There are only a few reports on environmentally friendly synthesis of metal NPs and their use^11^,^12^,^13^. Since the purification and separation of products require complicated procedures, simple processes for preparing NPs have been explored.

Our approach to addressing this issue is based on physical method. We have developed a matrix-sputtering method^14^,^15^,^16^,^17^,^18^, which is a simple technique for preparing metal NPs without formation of by-products. This method is based on vacuum metal evaporation using a liquid matrix with a low vapour pressure. In the sputtering process, after argon is ionized by high voltage, it attacks the target metal and ejects the target atoms or small clusters in a vacuum chamber. The ejected atoms and clusters coalesce not only in the gas phase but also inside or at the interface of the liquid matrix, forming NPs. The first sputtering method reported for the synthesis of metal NPs used ionic liquids as a liquid matrix owing to their characteristics of non-volatilization and good stabilization for the surface of metal NPs^19^. The use of non-volatile liquids such as other kinds of ionic liquids^14^, liquid monomers^20^, and polymers^15^,^16^,^21^, including silicone oils^22^ were reported later. Although these investigations have enabled the synthesis of Au NPs with diameters of 1–20 nm, it is still impossible to systematically control the size of particles to within a few nanometers. Alloy Au-Ag NPs with controllable plasmonic absorption was also obtained through this technique^23^,^24^.

To this end, recently we developed novel methodology to precisely control the size as well as photophysical property of metal NPs by sputtering process. While previous approaches have relied on the viscosity of liquid matrix for controlling particle diameters, our new approach introduced mercaptans as a stabilizer, inspired from chemical methods. In the chemical synthesis of metal NPs, controlling the concentration ratio between metal ions and stabilizing reagents is a possible means of systematic size control.

In our sputtering process, the mercaptan molecules dissolve in liquid matrix or evaporate in the gas phase and thus prevent the aggregation and growth of NPs by the effective coordination according to their concentrations, and result in a systematic size control in a single nanometer order. We have already reported several examples for size-controlled synthesis of gold or silver NPs with thiolate ligands^16^,^17^,^18^,^22^. These matrix sputtering resulted in a formation of Au NPs that fluoresce in NIR region. Very interestingly, Au NPs with diameters in a range of *ca.* 1.0–3.3 nm showed NIR fluorescence with a small spectral change according to their TEM diameters. These diameters are considerably larger than those reported for fluorescent Au clusters synthesized by chemical reduction method^25^,^26^. It should be noted that all samples had sorely uniform TEM size distribution histograms that match well to Gaussian fitting. Thus, in our previous paper^17^ we theorized that the obtained NPs via matrix sputtering are not single crystals, but instead are formed by the aggregation of very small, multinuclear Au complexes or clusters. However, NPs obtained by matrix sputtering method could not be purified well due to their less stability during purification processes and thus the detailed structural characterizations have not been achieved yet.

Hence, we here present a new matrix sputtering system for size-controlled synthesis of fluorescent Au NPs using 11-mercaptoundecanoic acid (MUA) as a stabilizing reagent. Long alkyl chain of MUA resulted in the formation of stable fluorescent Au NPs by sputtering. Thus, we could investigate detailed structural and photophysical characteristics of obtained Au NPs for the first time in order to discuss their unusual fluorescence mechanisms. We also investigated the effect of stirring and temperature of liquid matrix to structural and optical properties of the obtained Au NPs.

## Experimental Section

**Figure 1:**
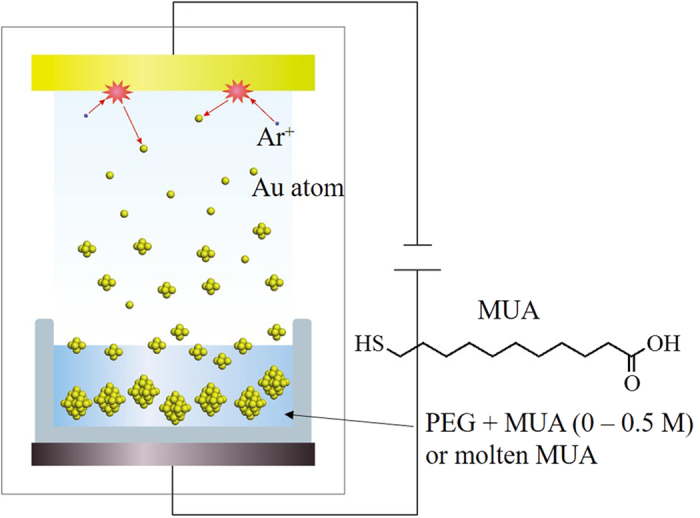
Schematic illustration of the matrix-sputtering method and chemical structure of MUA.

The experimental procedure is schematically illustrated in Fig. 1. First, to remove volatile substances, especially water, poly(ethylene glycol) (PEG) with molecular weight of 600 and MUA were dried under vacuum (rotary oil pump) at 100 °C for 2 h. The formation of bubbles in the vacuum chamber during sputtering, which may affect the sputtering rate and structure of the obtained NPs, was not observed. Next, 10 g of PEG and a corresponding amount of MUA were placed in a glass petri dish with a diameter of 6.5 cm and horizontally set against the sputtering target. The amount of MUA in PEG was set at 0, 10, 50, 100, 500, and 1000 mg (corresponding to 0, 5.2 × 10^−3^, 2.6 × 10^−2^, 5.2 × 10^−2^, 2.6 × 10^−1^, 5.2 × 10^−1^, and 5.2 × 10^−1^ M, respectively), and MUA completely dissolved in PEG under all conditions. In addition, sputtering into molten MUA at 50 °C (corresponding to 3.9 × 10^1^ M) was examined. Au was sputtered by a current of 20 mA under an Ar atmosphere at a pressure of 2.0 Pa. The distance between the surface of PEG and the surface of the Au target was set at 50 mm. Our unique sputtering system for NP preparation was equipped with a stainless stirring bar to stir the liquid matrix, which usually has a high viscosity. Sputtering was carried out for 20 min at room temperature under stirring at 100 rpm. To study the effect of stirring on the formation of Au NPs, we also investigated the sputtering of Au into PEG without stirring. Sputtering deposition at 50 °C of PEG solution containing MUA was also conducted. For the size fractionation experiment described in chapter 4, 60 min of sputtering deposition was carried out. Further experimental and characterization details are described in Supporting Information.

Obtained Au NPs showed very low dispersibility for common organic solvents, probably due to the multiple hydrogen bonds among carboxyl groups of MUA molecules on the NP surface. We purified by dissolving the Au NPs in dimethyl sulfoxide (DMSO) and re-precipitated the Au NPs in chloroform and ethanol. Since the fluorescence spectrum did not change during the purification, we concluded that we successfully purified the Au NPs and removed excess PEG and MUA by this procedure. The resultant Au NPs powder was then fractionated by HPLC (Jasco, LC-NetII/ADC system) according to their size. Size exclusion chromatography (SEC) column (Shodex, Asahipak GF-210 HQ, 300 × 7.5 mm, particle size of 5 μm) was used with the HPLC apparatus. Au NPs dispersion in DMSO (20 μL, concentration of 8.0 g L^−1^) was injected to the HPLC system. The mobile phase used was 100% DMSO and had a flow rate of 0.1 mL min^−1^ (Jasco, PU-4180) at a constant temperature of 40 °C (Jasco, CO-4060). The chromatogram was collected by measuring the absorption at 300 nm (Jasco, UV-4075), which is the excitation maximum of obtained fluorescent Au NPs.

## Results and Discussion

### Au sputtering into PEG: effect of stirring the liquid matrix on the size and uniformity of Au NPs

We investigated the preparation of Au NPs by a simple sputtering process with a magnetron-sputtering apparatus that was specially designed for NP preparation. The mechanism of this process is based on the discharge of a magnetron in which the electron field and magnetic field were located perpendicular to each other. We introduced Ar gas at 2.0 Pa to obtain homogeneous discharge. The value of this pressure, 2.0 Pa, was determined by the fact that the formation of a plasma during sputtering is not stable when the pressure is too low (e.g., 0.5 Pa). It should be noted that the deposition of a thin Au film on the liquid PEG was not observed in all experiments presented in this manuscript, while it usually forms on solid surfaces in SEM measurements.

**Figure 2:**
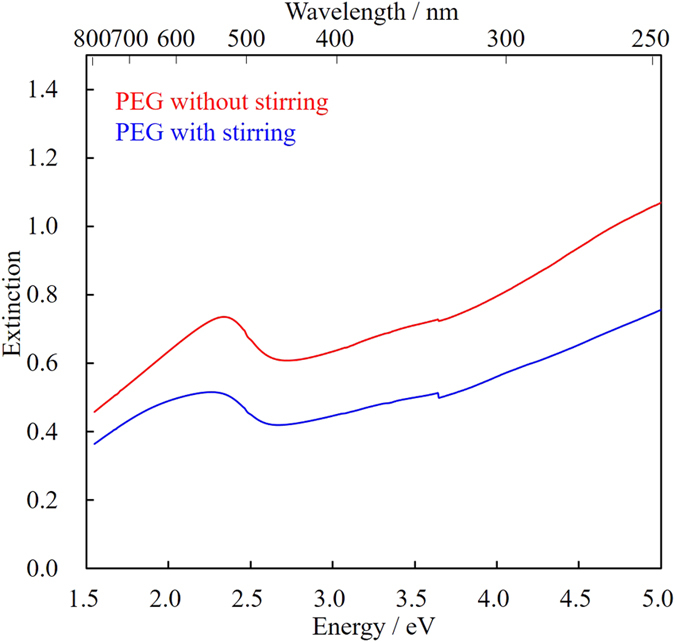
Extinction spectra of Au NPs prepared in PEG with and without stirring. The spectra were measured in a 1-mm quartz cell, without purification or dilution of the samples.

First, sputtering of Au into PEG was examined without mercaptans. To investigate the effect of stirring the viscous liquid matrix (PEG), our sputtering apparatus was equipped with a stainless stirring bar and stirred at 100 rpm. The extinction spectra of the dispersion of Au NPs in PEG were measured in a quartz cell with a 1-mm optical path just after the sputtering preparation, without further purification or dilution (Fig. 2). Broad absorption peaks around 2.4 eV (520 nm), which correspond to the localized surface plasmon absorption of Au NPs, were observed in the spectra of both samples prepared with and without stirring. However, the scattering of Au NPs drastically decreased with stirring while other sputtering conditions such as current and time (corresponding to the total number of sputtered Au atoms) remained the same. To investigate the detailed mechanism of this phenomenon, transmission electron micrographs were obtained.

**Figure 3:**
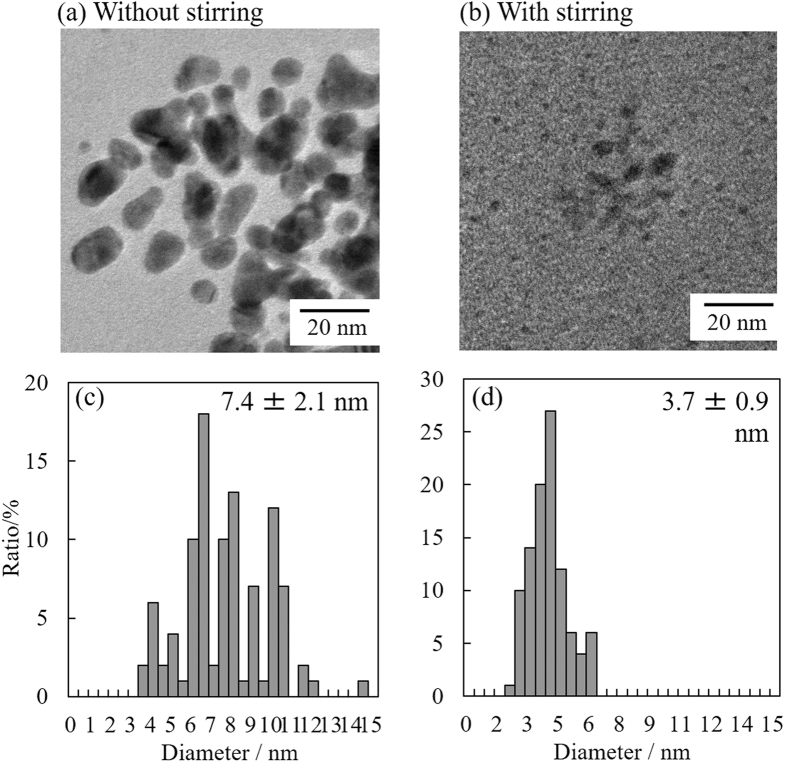
TEM images and size-distribution histograms of Au NPs prepared in PEG (**a,c**) without and (**b,d**) with stirring.

Figure 3 shows representative TEM images and size-distribution histograms of Au NPs prepared in PEG without and with stirring. The average sizes of Au NPs prepared without and with stirring were 7.4 ± 2.1 and 3.7 ± 0.9 nm, respectively. Based on these values, it is obvious that stirring of the liquid matrix decreased the particle size and yielded a rather uniform size distribution. It is important to note that the coalescence of nanoparticles on the surface of a viscous liquid matrix can be suppressed by stirring. Recently, the formation mechanism of metal NPs through a sputtering process has been investigated. It was found that the Au NPs first coalesce on the surface of the liquid matrix, and then further coalesce and grow inside the liquid^21^,^27^. In the previous study, these coalescence processes were controlled by the concentration of Au NPs and viscosity of the liquid matrix (PEG), which affected the collision probability of Au NPs inside or at the surface of PEG^21^. Without stirring, the average diameter of the resulting Au NPs (7.4 nm) was twice that of Au NPs obtained with stirring (3.7 nm). Thus, it can be considered that a continuous deposition of formed Au NPs on PEG, which has been considered a main reason of the growth NPs, was effectively suppressed by stirring. In other words, stirring continuously revealed fresh PEG surface during sputtering and a quick diffusion of Au NPs into the inside of liquid matrix was achieved, thus effectively suppressing further coalescence on the PEG surface. Moreover, it is considered that the stirring-related decrease in scattering in the entire wavelength region (Fig. 2) should be attributed to the decrease in particle diameter. It is well known that the absorption maxima of gold’s plasmon shifts to the red end of the spectrum as the diameter of the particles increases^28^. However, spectral shift occurs among particles with diameters above 20 nm and the shift is negligible for particles with diameters below 20 nm, as reported in a previous paper^28^. Thus, such red-shift due to the change in particle size was not observed in the current small NP system.

**Figure 4:**
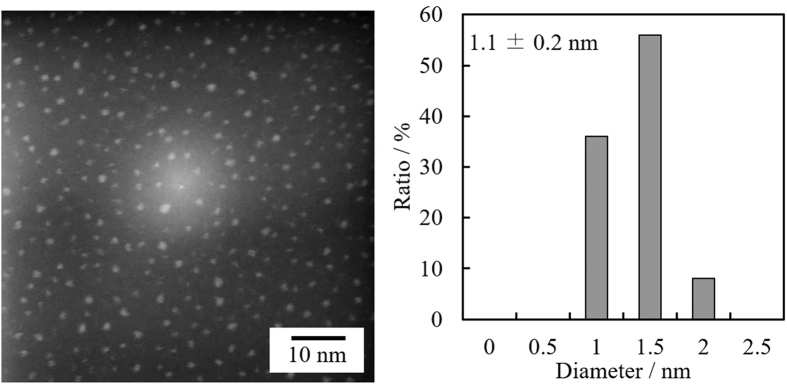
HAADF–STEM image and size-distribution histogram of Au NPs directly deposited on a carbon-coated TEM grid.

It is generally considered that the size of metal NPs that reach the surface of a liquid matrix or solid surface by sputtering is around 1–2 nm (although the precise dimension depends on the type of metal, sputtering current, and the distance between the metal target and liquid surface)^29^. To quantitatively examine the size of the NPs, we analyzed them by high-angle annular dark-field scanning transmission electron microscopy (HAADF–STEM). This HAADF–STEM method has significant advantages, including a realization of chemical contrast according to the atomic number (*Z*). Thus, a very small heavy-metal nanocomposite such as our Au NPs can be easily observed by HAADF–STEM. On the other hand, since the bright-field (BF)–STEM method depends on the decrease in the transmitted electron-beam intensity through scattering by the sample, the imaging on the atomic level is not very accurate. Therefore, in the current experiment, we examined the HAADF–STEM images to determine the size of directly sputtered small Au NPs. A sample for HAADF–STEM was prepared as follows (see a schematic illustration of the experimental setup in Figure S1, Supporting Information). A carbon-coated TEM grid was placed at the center of a petri dish and the distance between the Au target and the TEM grid was set at 50 mm, which is the same as the distance between the target and the surface of PEG in the experiments on Au NP preparation. Under the same current and chamber pressure, Au sputtering was preformed with a shutter, which can completely stop the approach of NPs formed outside to obtain a stable plasma, because the initial ignition of plasma was unstable. The shutter was then opened for approximately 0.5 s to deposit only a small amount of Au NPs onto the TEM grid. This procedure was designed to eliminate the high-density deposition of Au NPs onto the grid and thus allowed us to neglect the coalescence of NPs on the grid. This situation also enabled us to eliminate the risk of coalescence and growth of Au NPs during the electron-beam irradiation. A HAADF–STEM image of the obtained sample is shown in Fig. 4 (see also the BF–STEM image in Figure S2).

The bright spots in the HAADF–STEM image represent the Au NPs, and the average diameter was determined to be 1.1 ± 0.2 nm. The uniform distribution and sufficiently low density of NPs confirm that the obtained average diameter reflects the real diameter of NPs that reached the surface of the TEM grid. Moreover, the same value was obtained from the BF–STEM image (Figure S2), further supporting the HAADF–STEM result. These findings indicate that the coalescence of nanoparticles was primarily facilitated on the surface of the liquid matrix in the matrix-sputtering method. The main reason the average diameter of Au NPs prepared in PEG with stirring (3.7 nm) was more than three times larger than that of Au NPs in the control experiment (1.1 nm) should be the coalescence of nanoparticles at the surface of the liquid PEG or inside the liquid, even when a stirring bar was used, because PEG has a high viscosity and does not have any effective functional groups such as mercapto groups or amine groups to stabilize the Au NPs^21^. This is the first determination of the size of initial sputtered gold nanoparticles by using high resolution STEM.

### Au sputtering into PEG containing MUA: effect of MUA concentration on the size and photophysical characteristics of Au NPs

**Figure 5:**
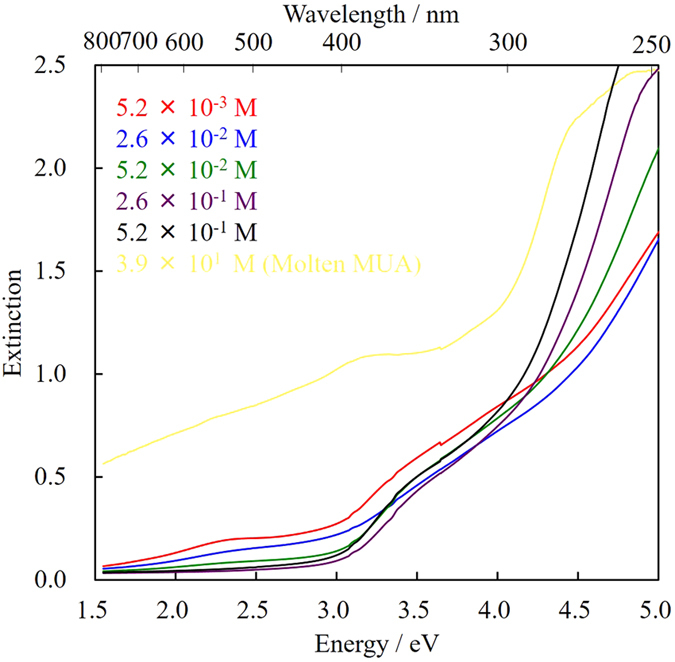
Extinction spectra of Au NPs prepared in PEG with various concentrations of MUA. The spectra were measured in a 1-mm quartz cell without dilution and purification of the samples (the sample sputtered into molten MUA was diluted with 10 times the amount of methanol (w/w) for the measurements).

To precisely control the diameter of Au NPs by sputtering, organic mercaptan was added to the liquid matrix, PEG, to coordinate it with the surface of NPs. The presence of mercaptan should suppress the coalescence of nanoparticles on or in PEG, and it was expected that the size of the obtained Au NPs could be controlled by adjusting the concentration of mercaptan. Figure 5 shows extinction spectra of Au NPs prepared in PEG with various concentrations (5.2 × 10^−3^ to 5.2 × 10^−1^ M) of MUA, and Au NPs prepared directly from molten MUA (corresponding to 3.9 × 10^1^ M). MUA is also non-volatile and can be easily and homogeneously dissolved in PEG. The sample sputtered into molten MUA was diluted with 10 times (w/w) as much methanol for the extinction measurements. Since the concentration of MUA in the sputtered sample prepared from molten MUA in methanol was too high compared with other samples, the extinction spectrum (yellow line in Fig. 5) shows much scattering and absorption by MUA. The surface plasmon absorption at 2.4 eV, which was also observed in Fig. 2, drastically decreased as the MUA concentration increased and almost disappeared when the concentration exceeded 5.2 × 10^−2^ M. Surface plasmon absorption originates from the vibration of a group of free electrons in the surface region of NPs, and they show an absorption peak corresponding to their vibration frequency. Therefore, the generation of plasmon absorption requires a certain particle size (~2.4 nm for Au NPs based on a previous report)^7^,^30^,^31^. Judging from the absorption spectra in Fig. 5, the size of Au NPs should decrease with increasing MUA concentration; the diameter of Au NPs prepared from a higher MUA concentration should be very small and thus no plasmon absorption was observed. At a MUA concentration above 5.2 × 10^−2^ M, a new absorption shoulder was observed at around 3.5 eV instead of the plasmon absorption. This absorption may be attributed to the small Au NPs^30^, as reported in our previous paper^17^.

**Figure 6:**
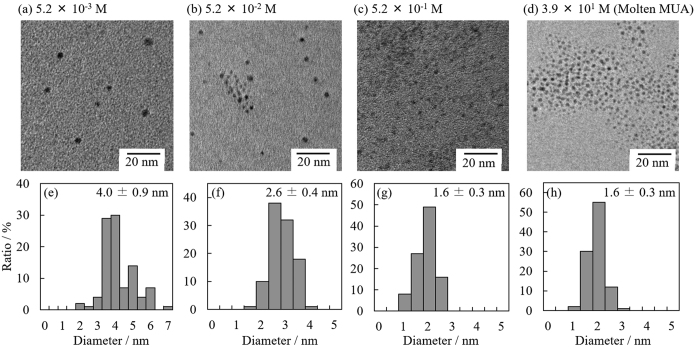
TEM images and size-distribution histograms of Au NPs prepared in PEG with various concentrations of MUA: (**a,e**) 5.2 × 10^−3^ M; (**b,f**) 5.2 × 10^−2^ M; (**c,g**) 5.2 × 10^−1^ M; (**d,h**) 3.9 × 10^1^ M (molten MUA).

Figure 6 shows representative TEM images and size-distribution histograms of Au NPs prepared in PEG with various concentrations of MUA. The average size of Au NPs decreased according to the MUA concentration, and the distribution histograms agree with the Gaussian distribution. Compared to the NPs prepared without MUA, these rather uniform size distributions (Fig. 3) were achieved by adding MUA to PEG to suppress the coalescence of nanoparticles on or in PEG.

**Figure 7:**
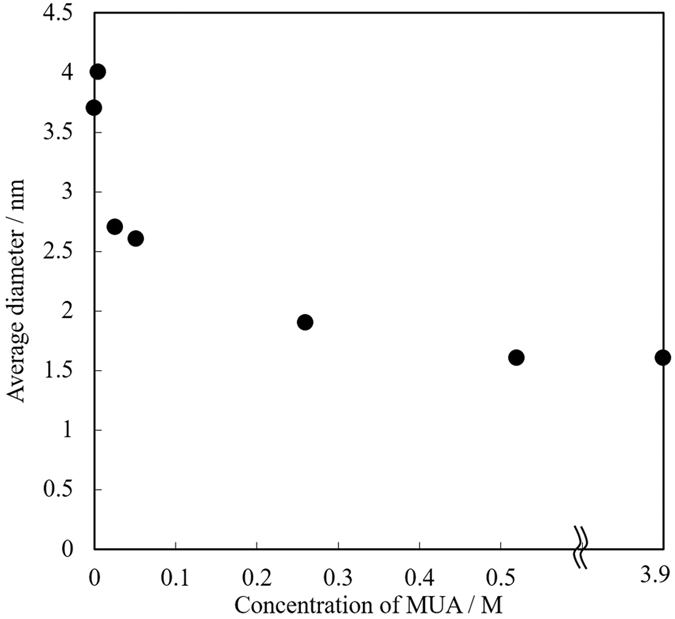
Size of Au NPs prepared in PEG with various concentrations of MUA (0 to 5.2 × 10^−1^ M). The data point at 3.9 M corresponds to Au NPs prepared in molten MUA.

The change in average particle size as a function of MUA concentration is shown in Fig. 7. It is obvious that higher MUA concentration generated smaller Au NPs, which can be simply explained by the collision probability of MUA with Au NPs inside and at the interface of PEG. Moreover, the difference in their particle sizes reflects the change in plasmon absorption in Fig. 5. Thus, this result clearly indicates that, as expected, the size of Au NPs can be controlled by the concentration of mercaptan-stabilizer in the liquid matrix.

The average diameters of Au NPs prepared in PEG with 5.2 × 10^−1^ and 3.9 × 10^1^ M (molten MUA) of MUA were the same (1.6 nm), indicating that the effect of MUA on the particle diameter had reached its limit under the current condition. It was estimated that the concentration of MUA was high enough to stabilize the nanoparticles, but the NP diameter was considerably larger than that of Au NPs that reached the surface of PEG (1.1 nm, determined from the HAADF–STEM image in Fig. 4). The most plausible reason for this difference was a slow diffusion^27^ of deposited Au NPs owing to the high viscosity of PEG, indicating the continuously deposited Au NPs slightly coalesced on the surface of PEG before they were completely stabilized by MUA.

Additional sputtering experiment was conducted under lower viscosity condition by increasing temperature (50 °C) with 5.2 × 10^−1^ M of MUA solution in PEG. TEM diameter of obtained Au NPs was same (1.6 nm, Figure S4) as that prepared at r.t. (Fig. 6). The photophysical property (described later) was also constant (Figure S4). Thus, we concluded that the temperature of PEG, which controls the viscosity, does not strongly affect nanoparticle formation under high concentration of MUA, while the drastic effect of viscosity in nanoparticle diameter has been observed in pure PEG or ionic liquids systems^21^,^29^. Detailed discussion on the effect of viscosity on the nanoparticle growth mechanism in thiolate matrix has been reported in our recent paper^32^.

**Figure 8:**
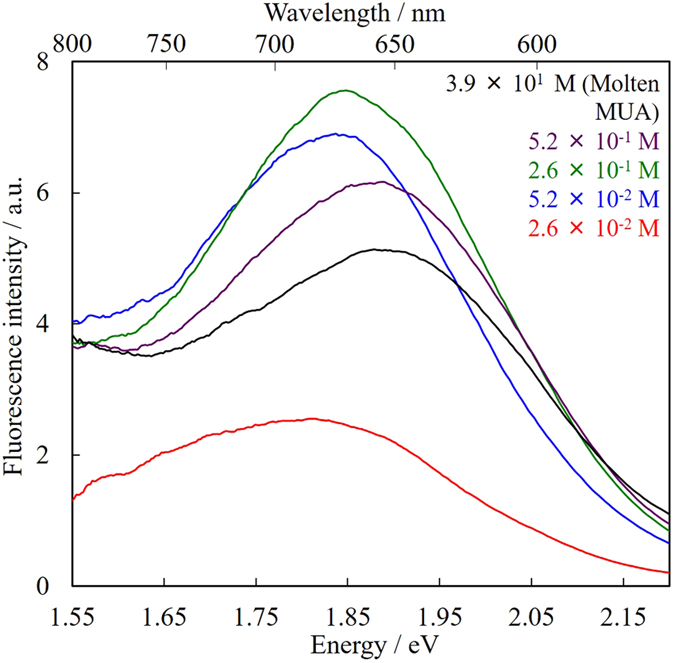
Fluorescence spectra of Au NPs prepared in PEG with various concentrations of MUA. The excitation wavelength was set at 300 nm. The sample sputtered into molten MUA was diluted with 10 times the amount of methanol (w/w) for the measurements.

The absence of plasmon absorption of obtained smaller Au NPs allowed us to measure fluorescence spectra. The excitation wavelength was set at 300 nm. The Au NPs prepared at 0 and 5.2 × 10^−3^ M of MUA, which showed plasmon absorption (Figs 2 and 5), did not fluoresce. On the other hand, those prepared in PEG with 2.6 × 10^−2^ to 3.9 × 10^1^ M of MUA exhibited fluorescence, as shown in Fig. 8. Although the spectral shapes were similar, however, the fluorescence maxima slightly shifted to longer wavelengths according to the MUA concentration. As summarized in Table 1, the trend of the fluorescence red-shift appears to be correlated with the average particle size. In other words, larger NPs prepared in PEG with lower MUA concentration tended to show fluorescence in longer-wavelength regions. This phenomenon has already been observed in our previous sputtering systems, and we have concluded that these differences in fluorescence maxima can be explained by the quantum size effect^33^; where the band gap of NPs depends on the number of atoms making up the particle and the red-shift in fluorescence corresponds to their sizes^34^. Fluorescence quantum yields were measured by an absolute method. The obtained values are summarized in Table 1, and all samples showed quantum yields of around 1%. This value is similar to those of fluorescent gold nanoclusters prepared by various synthetic methods including the sputtering method and the chemical-reduction method^35^,^36^. Compared with the quantum yield data of our previous alpha-thioglycerol (α-TG) capped Au NPs, which had very high quantum yield (maximum 16%)^17^, the quantum yields of MUA-stabilized ones were much smaller (~1%). The difference between the quantum yields of these two fluorescent Au NPs might originate from the preparation procedure. α-TG was not dissolved in PEG, but placed separately in the chamber. Therefore, the capping of α-TG onto gold surface took place in the gas phase, while MUA stabilizes the NPs on the surface of liquid matrix. The difference of these stabilizing processes should affect the detailed structure of obtained NPs due to the mechanism as described below.

While we successfully obtained fluorescent Au NPs via sputtering in the present and previous methods, the mechanism of their fluorescence is still unknown. Typically, Au NPs with a diameter of 1.6 nm or above prepared by a chemical reduction method do not fluoresce in a visible region. It is well known that the fluorescence wavelength of such very small Au clusters depends on the number of Au atoms consisting the cluster. However, in the present (MUA) and previous matrix sputtering systems (alpha-thioglycerol (αTG)^17^ or pentaerythritol tetrakis(3-mercaptopropionate)^15^ as stabilizers), Au NPs with diameter in a range of *ca.* 1.0–3.3 nm showed NIR fluorescence with a small spectral change (from 657 nm to around 750 nm). Some of these diameters (especially > 2 nm) are significantly larger than that reported previously for fluorescent Au clusters, and the range should be large enough to exhibit more drastic spectral change in their fluorescence. It should be noted that all samples had sorely uniform TEM size distribution histograms that match well to Gaussian fitting. Thus, in our previous paper^17^ we theorized that the obtained Au NPs are not single crystals, but instead are formed by the aggregation of very small, multinuclear Au complexes or clusters. This is the most plausible reason for unusual photophysical properties of Au NPs in the matrix sputtering methods.

### Purification and detailed characterization of obtained fluorescent Au NPs

In order to reveal the mechanism of unusual photophysical characteristics, we tried purification of our Au NPs prepared via sputtering into 5.2 × 10^−1^ M MUA-PEG solution. Au NPs showed very low dispersibility for common organic solvents, probably due to the multiple hydrogen bonds between carboxyl groups of MUA. Finally we purified by dissolving the Au NPs in dimethyl sulfoxide (DMSO) and re-precipitated the Au NPs in chloroform and ethanol. The obtained yellow powder showed red emission under UV irradiation (Figure S5) and the fluorescence spectrum did not change during the purification. Thus, we concluded that we successfully purified the Au NPs and removed excess PEG and MUA by this procedure.

**Figure 9:**
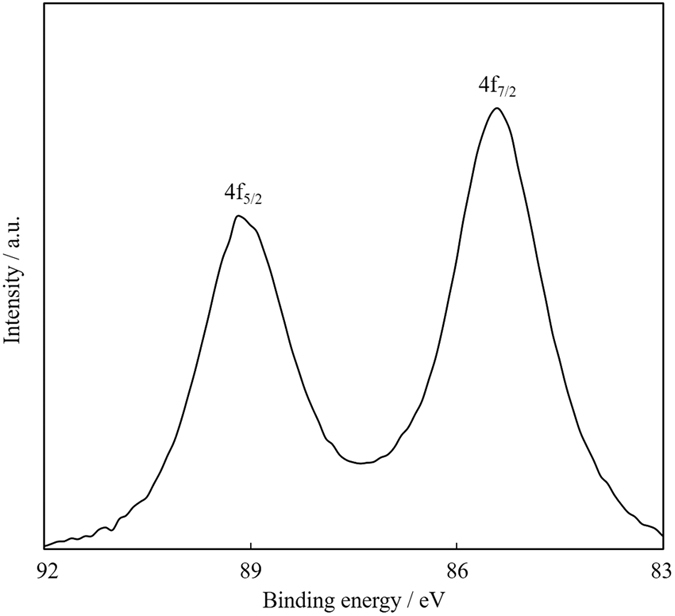
XPS spectrum of purified Au NPs for Au_4f_ region.

For this purified Au NPs, we first attempted to analyze XPS for Au_4f_ (experimental details and wide spectrum are shown in SI (Figure S6)). As shown in Fig. 9, we observed two peaks at 89.2 and 85.5 eV those correspond to 4f_5/2_ and 4f_7/2_, respectively. These peak positions are positively shifted from those of bulk gold (87.4 and 84.0). Tsukuda and co-workers have reported systematic synthesis and isolation of thiolate protected gold clusters^37^, and the positive XPS shift has been observed when the core cluster size decreased. Compared with their isolated products from Au_10_(SR)_10_ to Au_38_(SR)_24_ clusters, our XPS data matched well to those of a mixture of Au_10_(SR)_10_, Au_11_(SR)_11_ and Au_12_(SR)_12_, and isolated Au_15_(SR)_13_, where their peak maxima have been observed at 89.1 and 85.5 eV, respectively. Larger gold clusters such as Au_25_(SR)_18_ or Au_38_(SR)_24_ showed significantly different XPS maxima (88.2 and 84.8 eV) due to the decrease of surface Au atoms^37^. These results indicate that our Au NPs prepared by sputtering method contain lots of Au^+1^ atoms that are usually detected in very small gold clusters or gold-thiolate complexes, while the TEM diameter for our Au NPs was *ca.* 1.6 nm. Thus, XPS result strongly suggests that our Au NPs are not the single crystals and composed of the aggregates of very small species of clusters or complexes as already theorized for fluorescence mechanisms.

**Figure 10:**
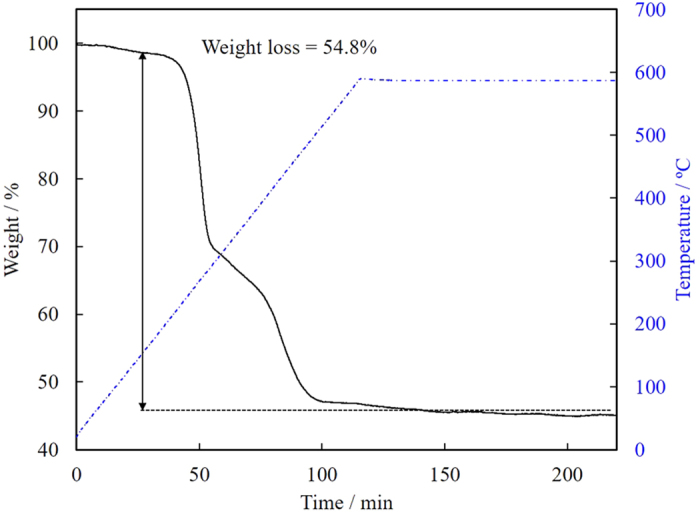
TGA curve (black line) of purified Au NPs under air at a flow rate of 100 mL min^−1^. Temperature (blue line) was increased from room temperature to 600 °C at a heating rate of 5 °C min^−1^ and then kept for 2 h.

TG analysis was carried out in order to determine the ratio between gold and thiolate ligand (generally expressed as Au/SR ratio). The obtained pure Au NPs was put into alumina cell, and the temperature was increased to 600 °C at hearting rate of 5 °C min^−1^ and then kept for 2 h under air flow. A slight decrease of weight below 150 °C is the evaporation of adsorbent water since MUA has a carboxylic acid group. As a result, we obtained Au/SR ratio of 0.96 by the calculation from the weight loss (54.8%, Fig. 10). This value is much smaller than that of Au_144_(SR)_60_ cluster (Au/SR = 2.4, the core diameter is 1.6 nm^38^; most consistent core diameter with our obtained Au NPs). Moreover, this Au/SR ratio closes to that of Au_12_(SR)_12_ or smaller clusters those have Au/SR ratio of 1 reported in a literature^37^, while the TEM diameter of our sample was significantly larger. Thus, as with the XPS result, this TGA result suggests that the obtained Au NPs are not single crystal, and consist much amount of thiolate ligands in their structure. These structural investigations strongly support the consideration for the mechanism of unusual fluorescence property as described above.

### Isolation of the obtained Au NPs by size-exclusion chromatography for understanding their unusual structural and photophysical characteristics

For the further understanding of nanoparticle property as well as nanoparticle formation mechanism via sputtering method, we attempted a systematic size fractionation of obtained fluorescent Au NPs. Since obtained Au NPs did not dissolve in common organic solvent except for DMSO, typical reverse-phase column chromatography was not applicable. Hence, size exclusion chromatography (SEC) equipped with a high performance liquid chromatography (HPLC) was carried out for the fractionation according to their size.

**Figure 11:**
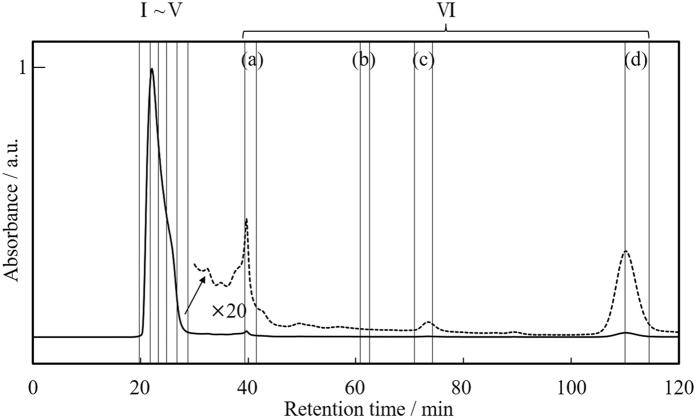
SEC-HPLC chromatogram of Au NPs detected by absorbance at 300 nm.

Au NPs prepared with 5.2 × 10^−1^ M of MUA (average TEM diameter of 1.6 ± 0.3 nm) was used for isolation. Figure 11 shows a chromatogram of Au NPs recorded by monitoring the absorbance at 300 nm that is the excitation maxima of fluorescence Au NPs. The chromatogram contains multiple peaks, indicating that a mixture was separated successfully by SEC-HPLC system. The relative intensity of each peak does not directly reflect the relative abundance of each component in the sample since the extinction coefficient at observed wavelength is unknown. Due to the very small amount for further analyses obtained after 30 min in chromatogram, we integrated the fractions (a–d) into one fraction (VI). We never observed any peaks after the fractionation for 120 min.

**Figure 12:**
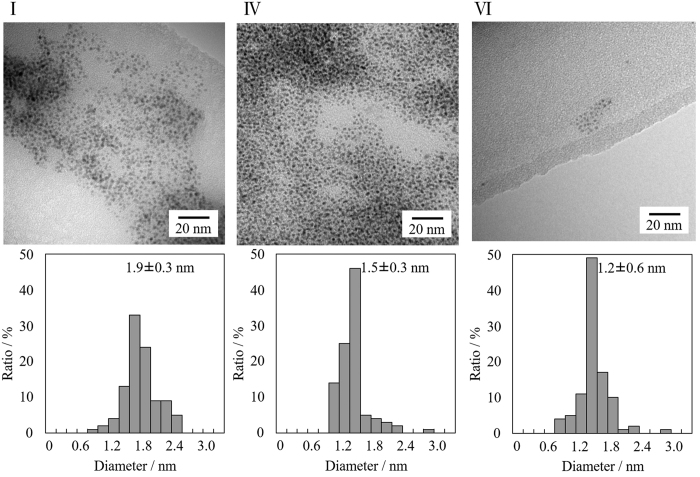
Representative TEM images and size distribution histograms of fractions I, IV and VI. For the histograms, more than 100 particles from several TEM images were counted.

Figure 12 shows representative TEM images and core size distributions obtained from these images of fractions I, IV and VI (those for other fractions are shown in Figure S7). All fractions have a narrow core size distribution and the average core diameters slightly decreased in the order from I to VI, as summarized in Table 2. The core diameters were successfully fractionated from 1.9 ± 0.3, 1.5 ± 0.3 and 1.2 ± 0.6 nm for fractions I, IV and VI, respectively, while the average core diameter before fractionation was 1.6 ± 0.3 nm (Fig. 6 and Table 1). These observations indicate that Au NPs have been successfully separated into different diameters by SEC-HPLC.

**Figure 13:**
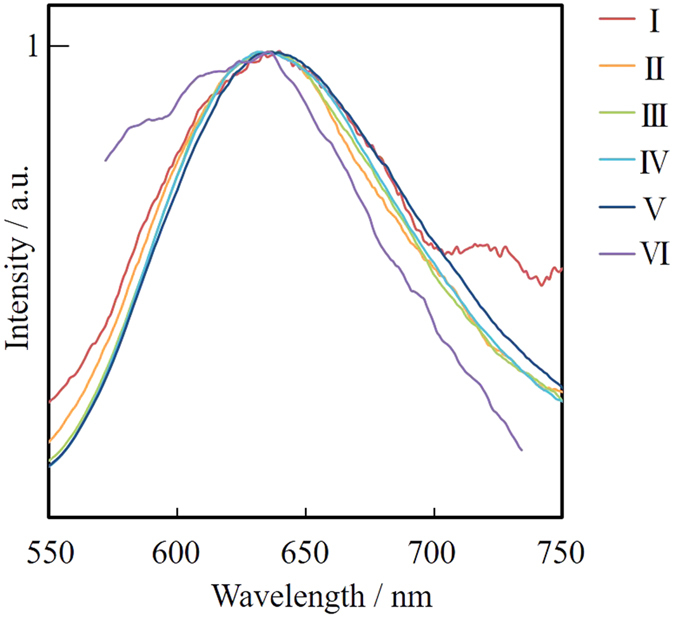
Normalized fluorescence spectra of fractions I to VI containing Au NPs in DMSO suspension excited at 300 nm.

Figure 13 shows fluorescence spectra for the Au NPs before fractionation, and fractions I-VI obtained by SEC-HPLC. Due to the very small concentration of VI, it was concentrated by the evaporation of DMSO solvent under 60 °C by spraying N_2_ gas. We never observed any change in TEM core diameters during the evaporation process. Very interestingly, we did not observe any spectral change in these fractions I-VI according to their core diameters (observed fluorescence maxima (λ_Fl_) are summarized in Table 2). Fluorescence of fraction VI included scattering due to the very tiny concentration. It is known that the fluorescence maxima tend to red shift as the number of atoms consisting the nanocluster increases according to the quantum size effect. While the difference in core diameters of fractions I-VI is certainly large enough to exhibit drastic spectral change in fluorescence, the observed fluorescence maxima were almost constant.

**Figure 14:**
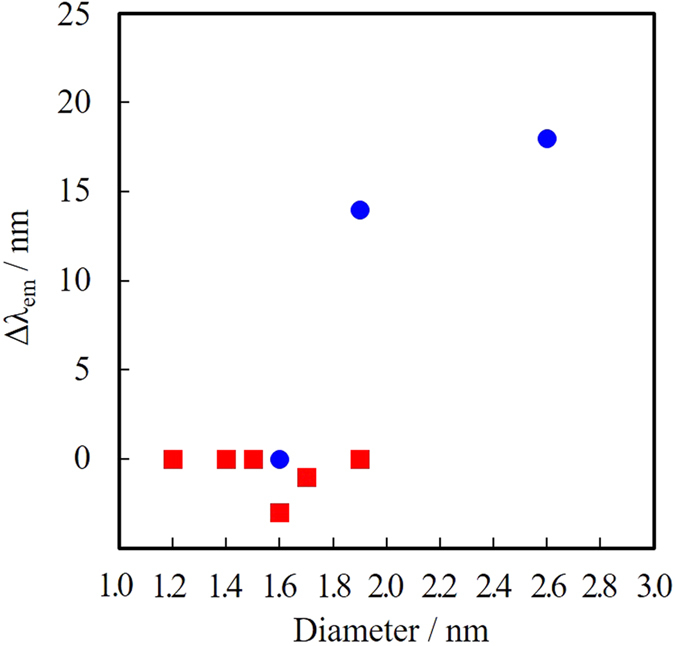
Correlation between TEM core diameters and fluorescence spectral change (Δλ_em_/nm). Red squares indicate the data from chapter 4 (fractions I to VI). Blue circles are data from chapter 2 where different sized fluorescent Au NPs have been synthesized by changing the concentration of thiolate ligand (MUA) during the sputtering deposition.

In order to discuss the mechanism of constant fluorescence maxima after the size fractionation, we compared the current data with chapter 2 (Table 1). In chapter 2, we synthesized MUA-stabilized Au NPs at various concentration of MUA in PEG. The average diameters of Au NPs could be systematically controlled from 1.6 nm to 2.7 nm those fluoresce in NIR region. Very importantly, Au NPs with different core diameters prepared at different MUA concentrations (blue circles in Fig. 14, data from Table 1 in chapter 2) showed obvious change in their fluorescence maxima. On the other hand, Au NPs with different core diameters fractionated from one synthetic batch in chapter 4 (red squares in Fig. 14. Δλ_em_: defined as the change of fluorescence maximum (in nm) from smallest core diameter) did not show such tendency. These results clearly suggest that the TEM core diameters do not determine fluorescence properties, however the synthetic protocol during sputtering deposition determines the photophysical properties of resultant NPs.

**Figure 15:**
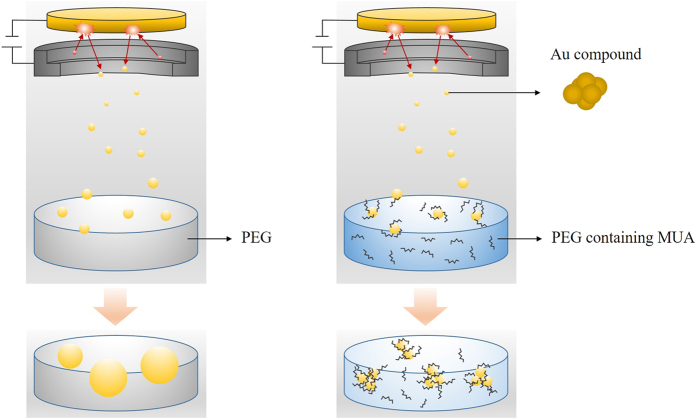
Plausible mechanism for the formation of Au NPs via sputtering deposition; (**a**) plasmonic Au NPs under the absence of MUA, (**b**) fluorescent Au NPs under the presence of MUA in PEG.

From these results, we here discuss the formation mechanism of our fluorescent Au NPs via sputtering deposition. First, under the absence of thiolate ligands (MUA), the sputtered Au atoms or clusters coalesce into larger clusters in the gas phase (as observed in Fig. 4), and further coalesce into plasmonic Au NPs on the surface and/or inside PEG (Fig. 3). The schematic illustration of this process is shown in Fig. 15a. On the other hand, Au NPs with unusual structural and fluorescent properties were prepared under the presence of MUA. Isolation studies in chapter 4 revealed that the TEM core diameters do not determine fluorescence properties, however the synthetic protocol during sputtering deposition determines the fluorescence properties of resultant NPs. These observations with structural characterizations described in chapter 3 clearly suggest that the obtained fluorescent Au NPs are not single crystals, but composed of the aggregate of smaller fluorescent cluster components. Thus, nanoparticle formation process under the presence of MUA can be depicted as shown in Fig. 15b. The coalescence process in a gas phase should be same to Fig. 15a, and then the clusters are quickly stabilized by MUA ligands on the surface of PEG with a very high concentration of MUA, resulting into a formation of quasi-stable clusters. These quasi-stable components further coalesce into larger Au NPs inside PEG, and finally result in a formation of fluorescent NPs with a TEM diameters of 1.6–2.7 nm (Fig. 6). Since the coalescence in PEG is a diffusion-limited process, the obtained Au NPs have a distribution in their diameters. However, the fluorescence wavelengths of these different sized Au NPs prepared at one MUA concentration were constant (Table 2) because were formed by the coalescence of smaller fluorescent clusters. Thus, isolation studies described in chapter 4 (Fig. 14 and Table 2) did not show any tendency between isolated particle diameter and fluorescence maxima.

Under various MUA concentrations in PEG, the fluorescence maxima shifted to longer wavelength as the particle diameter increased (Table 1). Different concentration of MUA should result in different stabilization rate, and in different fluorescent maxima due to different sized clusters. The deposited clusters on the PEG surface is very rapidly (slowly) stabilized by MUA under large (small) concentration of MUA, thus the fluorescence maxima tend to be at shorter (longer) wavelength. These discussions are the most plausible mechanism for the formation of fluorescent Au NPs via sputtering deposition.

Finally, we compare the current results with our previous system of αTG capped Au NPs^17^. Volatile α-TG was not dissolved in PEG and placed separately in the chamber during the sputtering of Au. While MUA stabilizes the NPs on the surface of liquid matrix (Fig. 15b), αTG stabilizes the ejected very small clusters rapidly in the gas phase. Moreover, almost negligible concentration of αTG in PEG matrix promoted the coalescence of these cluster components inside PEG matrix. Thus, αTG capped Au NPs also showed NIR fluorescence but their TEM diameters were relatively large (~3.3 nm).

## Conclusion

In conclusion, the synthesis of fluorescent gold nanoparticles (Au NPs) with controlled size in single nanometers was investigated by sputtering of Au into MUA-PEG solution. The size of the Au NPs was successfully controlled between 1.6 and 7.4 nm by regulating the MUA concentration. At MUA concentrations above 4 nm, surface plasmon absorption was observed, but it was not observed in smaller Au NPs. NIR fluorescence was observed in smaller Au NPs with around 1% of fluorescence quantum yield. The diameter (1.6–2.7 nm) was very strange to show such red emission compared with photophysical characteristics of reported gold clusters or nanoparticles synthesized by chemical method. By detailed investigations using TEM, HAADF-STEM, XPS, and TGA, and size fractionation technique using SEC-HPLC, we finally arrived at the plausible mechanism for the origin of unusual fluorescence property; the obtained gold nanoparticles are not single-crystal and are composed of aggregates of very small components such as multinuclear gold clusters or complexes. The reductants-free synthesis of Au NPs presented here represents a novel method to for the NPs that show unusual photophysical and structural characteristics.

## Additional Information

**How to cite this article**: Ishida, Y. *et al*. Thiolate–Protected Gold Nanoparticles Via Physical Approach: Unusual Structural and Photophysical Characteristics. *Sci. Rep.* **6**, 29928; doi: 10.1038/srep29928 (2016).

## Supplementary Material

Supplementary Information

## Figures and Tables

**Table 1:** Summary of the average diameter, maximum fluorescence wavelengths and fluorescence quantum yields for Au NPs prepared in PEG with various concentrations of MUA.

**Concentration of MUA/M**	**Average diameter/nm**	**Fluorescence maximum/nm**	**Fluorescence quantum yield**
2.6 × 10^−2^	2.7 ± 0.6	684	1.0%
5.2 × 10^−2^	2.6 ± 0.4	675	1.3%
2.6 × 10^−1^	1.9 ± 0.3	671	1.3%
5.2 × 10^−1^	1.6 ± 0.3	657	1.0%
3.9 × 10^1^*	1.6 ± 0.3	660	0.7%

*Sputtering into molten MUA.

**Table 2:** Summary of TEM core diameters and fluorescence maxima for fractions I to VI.

**Fraction**	**TEM diameter/nm**	**Fluorescence maximum/nm**
Mixture*	1.6 ± 0.3	636
I	1.9 ± 0.3	637
II	1.7 ± 0.4	636
III	1.6 ± 0.3	634
IV	1.5 ± 0.3	637
V	1.4 ± 0.3	637
VI	1.2 ± 0.6	637

Before fractionation by SEC-HPLC.
