# Supplementary material for: Thiolate–Protected Gold Nanoparticles Via Physical Approach: Unusual Structural and Photophysical Characteristics
Source: Sci Rep. 2016 Jul 18;6:29928. doi: 10.1038/srep29928 (PMC4947912; doi:10.1038/srep29928)
Supplement: Supplementary Information [file srep29928-s1.doc]

**Supporting Information for**

**Thiolate–Protected Gold Nanoparticles Via Physical Approach: Unusual Structural and Photophysical Characteristics**

Yohei Ishida, Ikumi Akita, Taiki Sumi, Masaki Matsubara, and Tetsu Yonezawa*

Division of Material Science and Engineering, Faculty of Engineering, Hokkaido University, Kita 13, Nishi 8, Kita-ku, Sapporo, Hokkaido 060-8628, Japan.

*Corresponding author E-mail address: tetsu@eng.hokudai.ac.jp

**Experimental details**

**Materials**

Polyethylene glycol (PEG) was purchased from Wako (Japan). Its average molecular weight was 600 and the degree of polymerization was between 10 and 17. 11-Mercaptoundecanoic acid (MUA) was purchased from Aldrich. Au target (99.9%) was supplied by Shinkuu Device (Ibaraki, Japan).

**Analysis**

UV-Vis extinction spectra were measured using a spectral photometer (Jasco, UV–630, 250 - 800 nm) with a quartz cell of 1 mm optical path, immediately after the sputtering deposition without dilution and purification. The Au NPs, which was prepared by the sputtering into molten MUA, was diluted with 10 times of methanol (w / w) for extinction and fluorescence measurements.

Emission and excitation spectra of the obtained Au NPs dispersion in PEG were measured using a fluorescence spectrophotometer (Jasco, FP-6600) with a quartz cell of 10 mm optical path. The filter (Sharp cut filter Y-50) which cut the emission less than 500 nm, was equipped in front of the detection window.

TEM observation for the size and shape of Au NPs was carried out using Hitachi H-9500 (acceleration voltage of 300 kV) or JEOL JEM-2000FX (acceleration voltage of 200 kV). TEM samples were prepared by dropping gold dispersion of PEG onto collodion-coated copper grids. The grids were then soaked into methanol for 30 min in order to remove the excess PEG and dried under vacuum.

STEM–HAADF and –BF observation was carried out using FEI TITAN3 G2 (acceleration voltage of 300 kV).

Fluorescence quantum yields were recorded with a Hamamatsu Quantarus-QY C11347-01 quantum yield analyzer with a quartz sample cell of 10 mm optical path. The measurement was carried out with a fixed excitation wavelength of each sample. The fluorescent wavelength was measured in the range of 200~950 nm. Au nanoparticle dispersion was used after diluted 5-fold with pure PEG.

XPS was detected by JEOL JPS-9200 X-ray photoelectron spectroscopy device equipped with a monochromatic Mg Kα source operating at 100 W under ultrahigh vacuum (~ 1.0 × 10^–7^ Pa) conditions. Binding energies were referenced to the C_1s_ binding energy of the adventitious carbon contamination.

TG-DTA was carried out using thermogravimetry analyzer (TGA, Shimadzu DTG­-60H). Measurement was operated using air at a flow rate of 100 mL min^–1^; and temperature was increased from room temperature to 600 °C at a heating rate of 5 °C min^–1^.

Figure S1. Schematic illustration of the experimental setup for sputtering onto TEM grid put on the center of petri dish. The distance between Au target and TEM grid was set at 50 mm.

Figure S2. BF-STEM image and size–distribution histogram of Au NPs deposited directly on a carbon-coated TEM grid.

Figure S3. Electric pictures of the samples under sunlight and UV light.


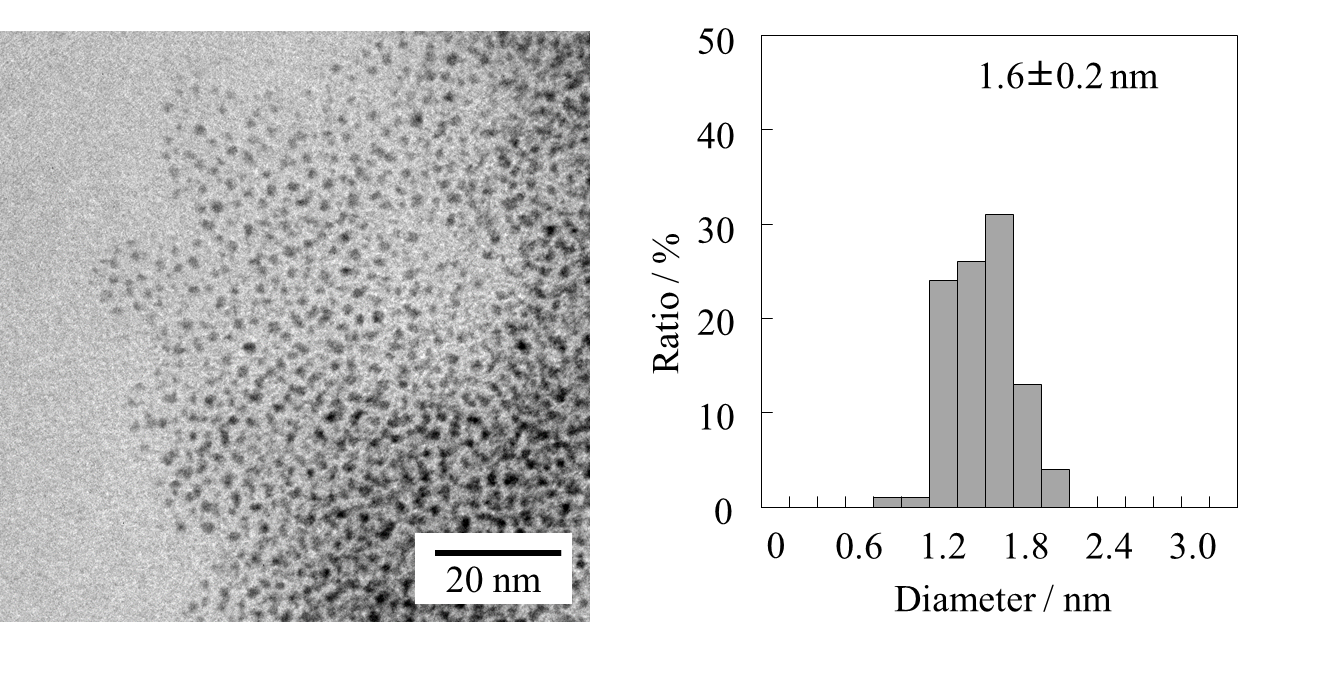

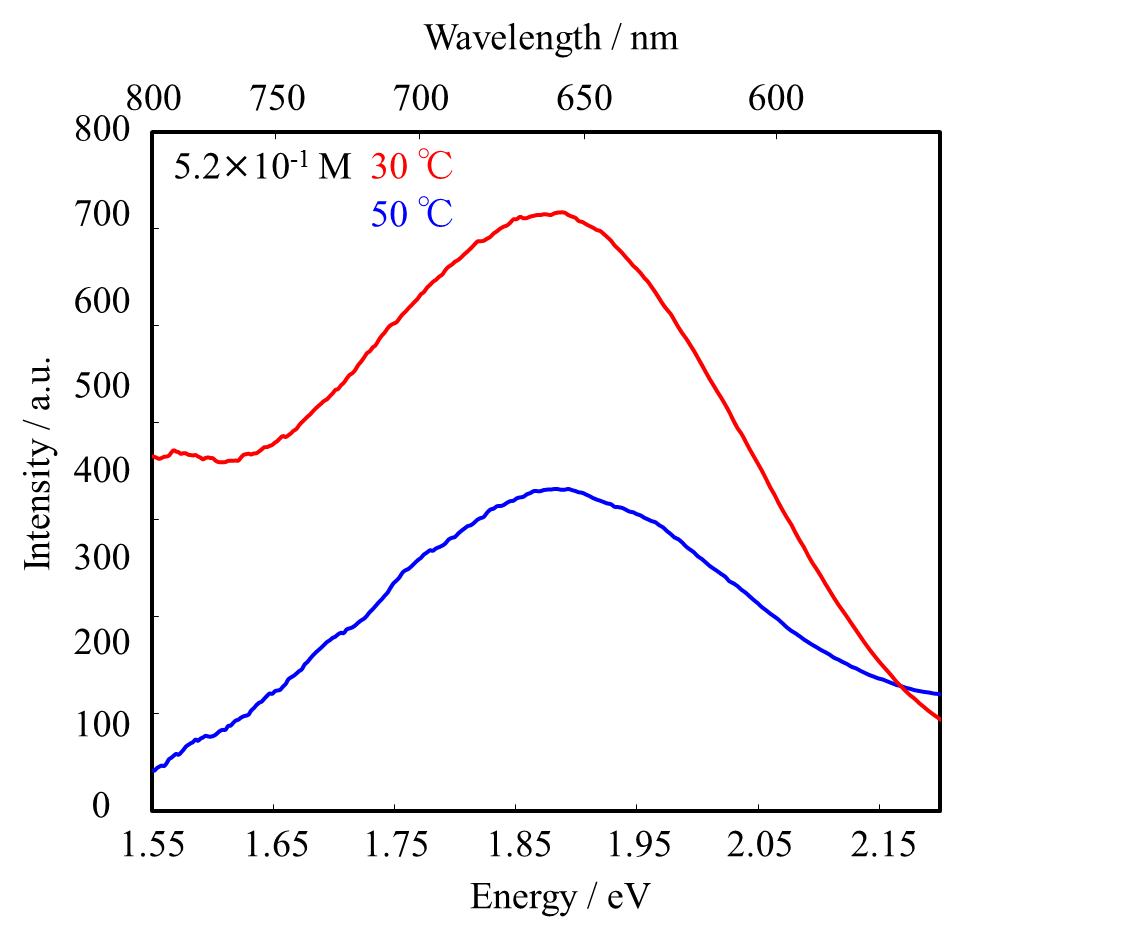


Figure S4. Fluorescence spectra of Au NPs dispersions in PEG containing 5.2 × 10^-2^ M of MUA prepared at r.t. and 50 ºC. TEM image and size distribution histogram of Au NPs prepared at 50 ºC. The average diameter (1.6 nm) was same to that prepared under r.t.

Figure S5. Electric pictures of the purified sample under sunlight and UV light.

Figure S6. Survey scan of XP spectrum for the purified Au NPs shown in Figure S5.


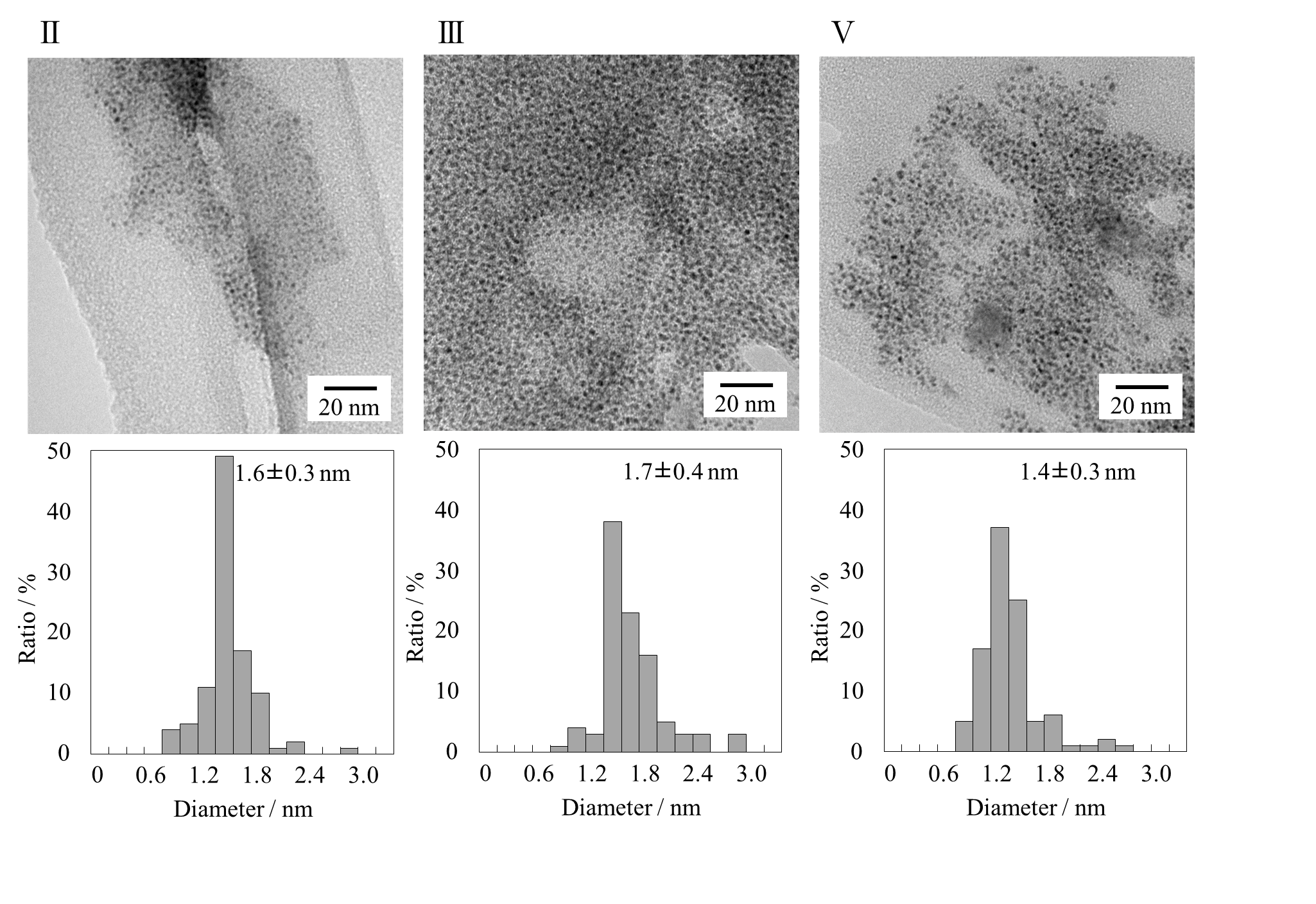


Figure S7. Representative TEM images and size distribution histograms of fractions II, III and V. For the histograms, more than 100 particles from several TEM images were counted.
